# Machine learning analysis of the effects of COVID-19 on migration patterns

**DOI:** 10.1038/s41598-024-80841-0

**Published:** 2024-11-30

**Authors:** Farzona Mukhamedova, Ivan Tyukin

**Affiliations:** King’s College London, London, WC2 R2LS UK

**Keywords:** Applied mathematics, Statistics, Scientific data, Environmental impact

## Abstract

This study investigates the impact of the COVID-19 pandemic on European tourist mobility patterns from 2019 to 2021 by conceptualizing countries as monomers emitting radiation to model and analyze their patterns through the lens of socio-economics and machine learning. By incorporating perturbations into clustering, this work evaluates the stability of mobility flux clustering under variable conditions, offering insights into the dynamics of socio-economic corridors. The results highlight distinct shifts in tourist behavior, with bimodal clustering in 2019 reflecting heterogeneous mobility patterns, whereas unimodal distributions in 2020 and 2021 indicate increased global uniformity, driven by pandemic-induced restrictions and gradual recovery. The PCA and dendrograms of the perturbed clustering reveal that tourist preferences align with GDP, cultural, and linguistic similarities, explaining regional cohesion and fragility. This study demonstrates the fragility of emerging socio-economic corridors like the Red Octopus compared to the resilience of established ones like the Blue Banana. The findings emphasize the importance of targeted policy interventions, such as strengthening transport infrastructure and fostering small and medium-sized enterprises (SMEs), to mitigate disruptions and promote balanced regional development. By integrating perturbations into clustering, this research introduces a strong framework for assessing mobility patterns under realistic variability to enhance economic resilience and anticipate shifts in tourist behavior during global crises.

## Introduction

Tourism is a cornerstone of many economies, particularly in Europe, where cross-border travel is commonplace. It plays a vital role in the global economy, contributing to cultural exchange, economic growth, and employment opportunities worldwide. The COVID-19 pandemic caused unprecedented disruptions to international travel, profoundly affecting tourist mobility patterns and challenging existing models of human movement. Understanding these changes is crucial for policymakers and businesses aiming to mitigate the economic impacts and plan for recovery. While studies on COVID-19 dynamics have largely focused on epidemiology, spatial predictors for the virus, and forecasting using neural networks and SARIMA models^1–5^, fewer have addressed the socio-economic impacts, particularly the fragility of economic corridors and mobility networks across regions^6–10^. There are also agent based systems such as Covasim^11^ and multi-scale data driven models to explain the transmission of COVID-19^12^ and mean-field ODEs^13^.

While these methods have been instrumental in understanding virus transmission, intervention strategies, and forecasting, they often focus on infection rates, clinical progression, and local transmission patterns. However, they generally fall short in addressing the broader socio-economic impacts, particularly how pandemics disrupt economic corridors and mobility networks across regions^6–10^.

Complex networks have emerged in various fields such as neuroscience^14^, the internet^15^, and transportation systems^16^. Within this framework, social networks offer critical insights into human geography, movement, history, and economic ties^17^. Studies on the structure of the global tourist network^17^ emphasize the importance of analyzing degree correlations, weights, and scale-free properties. However, these approaches rarely integrate the effects of perturbations, an essential consideration for understanding stability under variability or data inconsistencies.

Our work bridges the gap between socio-economics, applied mathematics and machine learning by adding to the discussion in understanding the dynamics of tourist mobility. It also lays a foundation for future explorations in geo-spatial and socio-economic modeling. One significant consideration in our study is treating countries as monomers emitting radiation, inspired by diffusion dynamics, to model mobility flux. This abstraction simplifies complex spatial interactions while capturing essential socio-economic characteristics such as GDP, cultural ties, and language similarities.

We establish a framework for evaluating the stability and resilience of the clustering observed. By using perturbation-enhanced clustering and PCA, we provide tools for anticipating shifts in mobility patterns and planning recovery strategies as we highlight the resilience and fragility of certain socio-economic corridors.

Perturbation-enhanced clustering, enables us to identify stable mobility patterns despite data inconsistencies or external disruptions. This addition addresses socio-economic imbalances, such as those evident in the Red Octopus corridor, while identifying specific clusters requiring targeted development to strengthen the European Union’s socio-economic networks. They are able to simulate variability in tourist behavior and data inconsistencies which allows them to provide a unique approach to uncover stable structural patterns. This approach allows room for extension to city urbanization projects.

Our work validates the opinion of Van der Meer^18^ in regards to the Red Octopus socio-economic corridor. This study illustrates regional cooperation is required due to the extreme fragility of the socio-economic corridors in Europe despite the efforts outlined in the Lisbon Strategy and European Cohesion Policy to name a few^8,19^. We provide a framework that informs strategic policy interventions and infrastructure investments. These findings contribute to broader socio-economic mobility studies, offering actionable insights to enhance resilience and cohesion across Europe’s economic corridors. By focusing on the structural changes in tourist mobility networks, our study goes beyond simple visitor forecasts and delves into the spatial and socio-economic inter-dependencies of tourism flows.

We show that economic resilience in the tourism sector can be strengthened through strategic partnerships and targeted investments, especially in regions disproportionately affected by disruptions in tourism. We uncover the underlying structural patterns in European tourism networks that allows us to reveal the impact of shared socio-economic characteristics on mobility pattern. We find connections to established socio-economic zones based on these socio-economic characteristics and we highlight the potential for targeted infrastructure investment and policy adjustments to elevate the entire corridor.

There are different theoretical perspectives to analyze the clusters and in tourism analysis it is clear from the perspective of tourism research that the three main types of benefits within the formation of tourism networks is learning and exchange, business activity, and community formation (see chapter 8 in^20^). Therefore it is important to observe and analyze the tourism network during the COVID-19 pandemic to extract important features (Table 1).

**Table 1:** Description of key parameters in the radiation model.

Parameter	Parameter description
$T_i$	Total number of tourists departing from location i
$T_j$	Total number of tourists departing from location j
$m_i$	Total population at location i
$n_j$	Total population at location j
$s_{ij}$	Total population in the circle of radius $r_{ij}$ centered at i
$s_{ji}$	Total population in the circle of radius $r_{ji}$ centered at j
N	Total number of locations

In order to proceed, an important model of interest from^21,22^ is also applied to describe the empirical data. The proposed is the radiation model, utilized by diffusion dynamics in the analysis of the trajectory of energetic particles or waves moving through a vacuum^23^. Nevertheless the radiation model is applicable in network science and more specifically in the application of human mobility and has been studied in regards to commuters, taxi drivers and job seekers between cities or states^24–26^. Therefore it is imperative to extend this notion to a larger scale and develop the radiation model and apply machine learning methods to evaluate the behavior of European tourists and the implications this has on policymakers.

Hence using the radiation model^27^, the predicted mobility flux from location *i* to location *j* is given as^28^,

1 $$\begin{aligned} <T_{ij}>= \frac{m_i n_j}{(m_i + s_{ij})(m_i + n_j + s_{ij})}. \end{aligned}$$

In the model, $r_{ij}$ has been defined as the radius of the circle centered at source *i* and with circle perimeter touching destination *j*. This is done so that the model can calculate the total number of individuals inside of this circle excluding the populations of both destination *j* and source *i*. With $r_{ij}$, the model then selects which countries to include in $s_{ij}$, where $r_{ik}$ is calculated for all countries *k* (excluding $k = i = j$) and if $r_{ik} < r_{ij}$ then the population of country *k* is included in the total $s_{ij}$,

$$\begin{aligned} s_{ij} = \sum _{k\in N_{ij}} m_k,\quad N_{ij} = \{k:r_{ik} < r_{ij}\} \end{aligned}$$

where $m_k$ is the population of location *k*.

In the calculation of $s_{ij}$, the assumption used is that if the capital city of a country *k* is within the radius $r_{ij}$ then the model includes the entire population of country *k* in $s_{ij}$, as opposed to directly working out the total number of people inside of the circle created by $r_{ij}$.

Recall that the adjacency matrix of a weighted network is a $N\times N$ matrix $\textbf{V}$ with elements:

2 $$\begin{aligned} V_{ij} ={\left\{ \begin{array}{ll} w_{ij}, & \text {node } \textit {j } \text{links to node } \textit{i } \text{with the link}\\ & \text {having a weight } w_{ij}\\ 0, & \text {otherwise}. \end{array}\right. } \end{aligned}$$

The adjacency matrix represents the connections between different countries where the row *i* and column *j* represent a different location with the value constituting the number of tourists entering *i* from *j*.

**Fig. 1:**
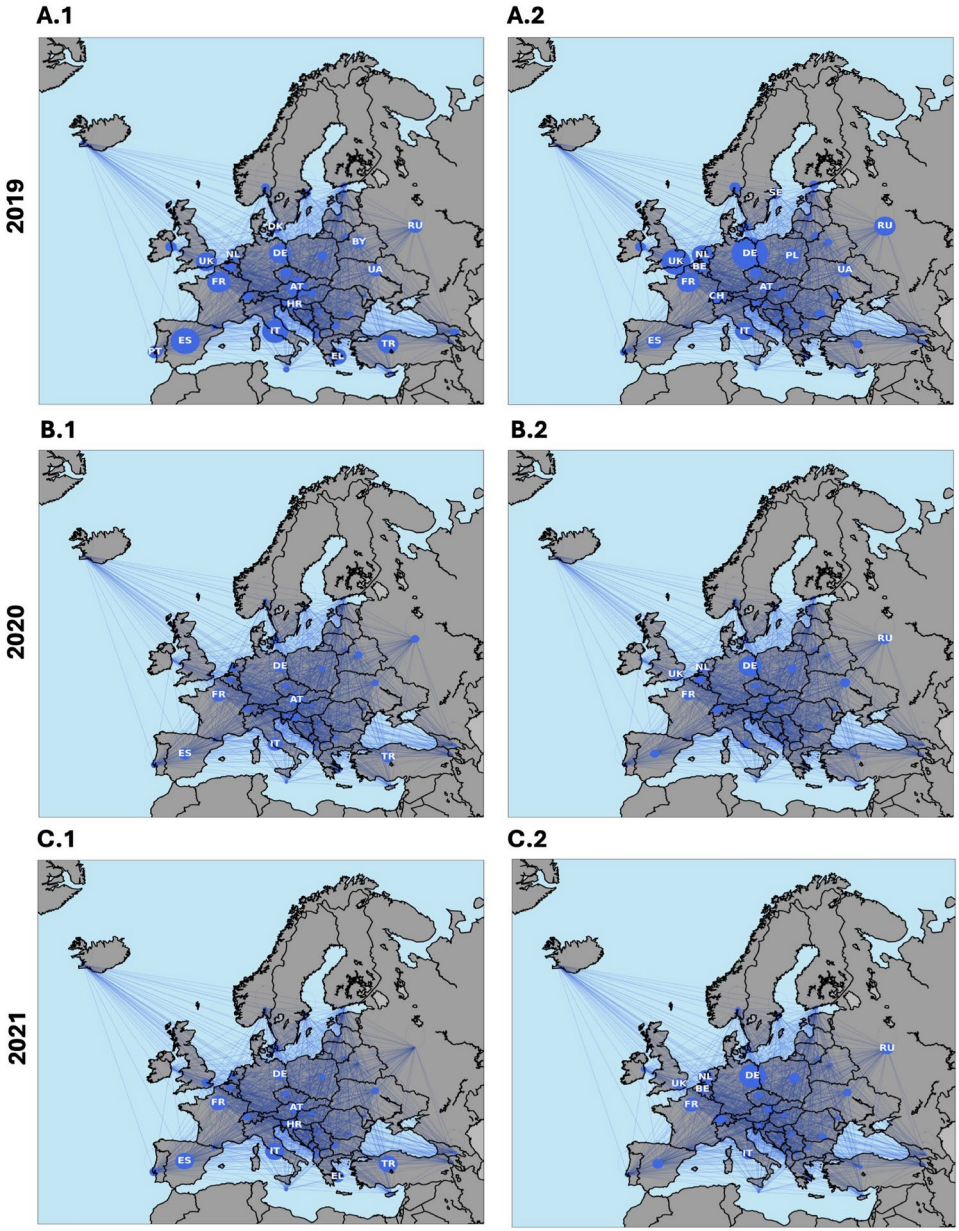
(**A.1–C.1**) The mobility flow going into the country given by the empirical data. (**A.2–C.2**) The mobility flow going out of the country given by the empirical data.(Produced using Python using packages networkx and Basemap.).

This destination model can be used to identify patterns and trends in tourism flows between different destinations, and to evaluate the impact of changes in tourism infrastructure or other factors on those flows^29,30^. By analyzing the adjacency matrix, tourism planners and policymakers can gain insights into the relationships between different destinations, pinpoint tourism hubs and develop strategies to promote and develop those destinations in a way that maximizes tourism flows^31,32^. These hubs can be visually observed from the empirical data seen in Fig. 1 where the main hubs are clearly defined after travel bans have been placed as well.

By observing this behaviour during a pandemic, tourist hubs, economic hubs and connections between countries become clear with casual travellers being filtered out naturally due to the travel bans placed during the pandemic. This allows us to identify areas that can strengthen tourism infrastructure, develop marketing strategies, develop economic ties and evaluate the clusters within the network which travellers tend to stay in.

## Methods

In the context of tourism flows, the similarity between the patterns of mobility flux between destinations are analyzed employing different similarity measures to compare the empirical data and the model. To validate the clustering Monte Carlo simulations introduce controlled perturbations to the distance matrices, allowing clustering performance to be evaluated across 100,000 scenarios. Principal Component Analysis (PCA)^33–35^ is then applied to reduce dimensionality while preserving variance, identifying the principal components that capture variability in the data and highlighting the structure of clusters. Additional details on data handling and the simulation protocol are provided in the Supplementary file to ensure transparency and reproducibility.

### Similarity between source and destination

Different methods measuring similarity are applied to the mobility flux matrix for the modeled data as well as the empirical data. The different metrics of measuring similarity are applied before the application of clustering algorithms to group similar countries together by reducing the complexity of the data.

Firstly the cosine similarity is applied as it measures the cosine of the angle between two vectors and is used frequently in natural language processing^36–39^ as it is a method that provides a strong way to differentiate images, datasets, etc. Hence the cosine similarity can help in identifying clusters of similar countries with respect to their tourist flow. Secondly, the Pearson correlation^40,41^, is also explored as it measures the linear correlation between two vectors, and is often used to measure the correlation between two variables which in this context measures the correlation between the patterns of tourism flows between countries. It is used to measure the strength of the relationship between two data points.

Moreover, the Manhattan distance measures the distance between two vectors by summing the absolute differences between their components which is significantly used in image processing^42,43^. The Euclidean distance measures the distance between two vectors by taking the square root of the sum of the squared differences between their components. These distance measures are used to evaluate the distances between the patterns of tourist flows between different countries.

Thus, by using different similarity measures to cluster, tourist flows can be identified from the modeled data and from empirical data. This can help tourism organizations and businesses better understand the patterns of tourist behavior and preferences. For example, countries with similar tourist flows may have similar cultural or economic ties, which could be useful information for marketing efforts. Similarly, identifying countries with dissimilar tourist flows could help identify opportunities for expanding tourism markets or developing new products or services.

### Unsupervised learning algorithms for clustering based on similarity measure

Then clustering can help identify patterns and trends in the data that may not be immediately apparent, allowing one to gain better insight into patterns and similarities that may not have been obvious. The methods employed with a combination of the mentioned similarity measures are: k-means clustering, Density-Based Spatial Clustering of Applications with Noise (DBSCAN) and hierarchical agglomerative clustering (HAC). Some of these methods can be seen in^44,45^ when applied to human mobility.

Note HAC creates a tree-like structure of nested clusters by iteratively merging the two closest clusters. This algorithm is useful for analyzing tourist flows because it can identify clusters of tourists at different levels of granularity. HAC can also handle different types of distance metrics and linkage methods, making it flexible and adaptable to different types of data to build a hierarchy of clusters.

In the context of HAC, the ward linkage method often performs the best because it minimizes the variance within each cluster and maximizes the variance between clusters. Other linkage criteria, such as single, complete, and average linkage exist, but the ward linkage typically produces the highest quality clustering results. Since the total within-cluster variance is minimized, it leads to more compact and well-separated clusters^46^. Therefore the distance between two clusters $C_i$ and $C_j$ is defined as the increase in total within-cluster variance after merging them. This is computed as,

3 $$\begin{aligned} D(C_i, C_j) = \frac{|C_i||C_j|}{|C_i| + |C_j|} \Vert \bar{x}_i - \bar{x}_j \Vert ^2. \end{aligned}$$

Meanwhile, DBSCAN is used to identify clusters of tourists that are concentrated in specific areas, k-means is useful for identifying *k* clusters of tourists that are similar in terms of their travel patterns, and HAC is well suited for identifying clusters of tourists at different levels of granularity as seen in Fig. 4.

To identify popular tourist destinations, DBSCAN may be the best choice as it can identify clusters of tourists that are concentrated in specific areas. On the other hand, if the goal is to identify different types of tourist flows, such as cultural tourism or adventure tourism, HAC may be the best choice (Table 2).

**Table 2:** Description of key parameters used in the ward linkage.

Parameter	Parameter description
$|C_i|$	Number of points departing in cluster and $C_i$
$|C_j|$	Number of points departing in cluster and $C_j$
$\bar{x}_i$	The centroids (mean vectors) of clusters $C_i$
$\bar{x}_j$	The centroids (mean vectors) of clusters $C_j$
$\Vert \bar{x}_i - \bar{x}_j\Vert$	The Euclidean distance between the centroids of the two clusters

Note that DBSCAN is a density-based clustering algorithm that groups together objects that are close to each other in a high-density area, while leaving out objects that are in low-density areas.This algorithm offers the ability to have no input from the user regarding the number of clusters and that it can identify clusters of tourists that are concentrated in specific areas^47^. However if the density of the data is not uniform it may not work as efficiently even if the algorithm is well suited to handling noise and outliers. Therefore it is imperative to compare results with other methods.

Lastly k-means clustering is also applied as it clumps the destinations into a fixed number (*k*) of clusters based on their similarity. Each destination is assigned to the nearest centroid. Then each centroid is based on the new assignments^48^. It is an iterative process that stops when the centroids no longer move or a maximum number of iterations is reached. Note, the Euclidean distance is normally used to calculated the distance between two points^49,50^. This algorithm is useful for analyzing tourist flows because it can identify *k* clusters of tourists that are similar to each other in terms of their travel patterns. However, note that k-means is sensitive to the choice of the initial centroids, and it may not work well if the clusters are not well-separated.

Other applications of the k-means clustering algorithm can be observed in customer segmentation, anomaly detection, etc^51^ since it offers computational efficiency in handling large datasets. However, due to the algorithm’s sensitivity to the initial placement of centroids whilst also specifying the number of clusters *k* in advance, other evaluations must be observed to identify *k* such as the elbow method (see accompanying code).

### Evaluation of the quality of the clustering assignments

The evaluation of the quality of clustering is undertaken in two steps: before the algorithm is implemented and after. Firstly the optimal number of clusters is chosen and then the quality of the resulting clusters are evaluated.

#### Choosing optimal number of clusters

Using the within-cluster sum of squares (WCSS)^52^ to measure the variability of the data within each cluster in a clustering algorithm, the sum of the squared distances between each country and its nearest cluster center is calculated. This method is referred to as the ‘elbow method’ to determine the optimal number of clusters in a dataset. The idea is to choose the number of clusters at the point of inflection of the curve, where the WCSS begins to level off. This indicates that adding more clusters does not result in a significant reduction in the sum of squares.

After the optimal *k* is chosen then the measure of how the cluster assignments is measured by measuring the silhouette score^53^ which ranges from -1 to 1 where a score close to 1 indicates better assignments where the country in the cluster is very similar to other countries within the cluster. Note a score of 0 indicates that the country sits on the border between clusters. Therefore the average silhouette score is taken to evaluate how well the empirical data and the simulated model data is clustered. The results can be seen in Table 3 especially for the empirical data where clearly using HAC Euclidean distances as a measure of similarity results in the best clustering when evaluating for 2 clusters for the years 2019 and 2021.

**Table 3:** The highest silhouette score achieved by the corresponding clustering method for each year for the data and model alongside the average silhouette score and the respective standard deviation from 100,000 Monte Carlo simulations where perturbations are added to the distance matrix.

Year	Data				Model			
	Clustering	Score	MC Score	MC $\sigma$	Clustering	Score	MC Score	MC $\sigma$
2019	Agglomerative Euclidean	0.743	0.419	0.065	Agglomerative Euclidean	0.769	0.428	0.093
2020	Agglomerative Manhattan k=2	0.786	0.463	0.021	Agglomerative Manhattan k=2	0.799	0.511	0.090
2021	Agglomerative Euclidean	0.841	0.529	0.017	Agglomerative Euclidean	0.784	0.430	0.039

### Principal component analysis

Principal Component Analysis (PCA) is employed to identify the principal components that maximize the variance in the dataset. This technique is crucial for reducing the dimensionality of the data while preserving the majority of the variance, thereby simplifying the complexity without significant loss of information.

The application of PCA to the mobility flux matrix results in a reduced-dimensionality representation, retaining the features that contribute most to the variance. This orthogonal set of principal components simplifies the data structure, facilitating easier analysis and visualization of clustering patterns. The principal directions of data variation, enhancing the comparison between empirical and modeled data.

The proportion of variance explained by each principal component is calculated as follows,

4 $$\begin{aligned} \text {Variance explained by a principal component} = \frac{\lambda _i}{\sum _{j=1}^p \lambda _j} \end{aligned}$$

where $\lambda _i$ is the eigenvalue of the *i*-th principal component, and *p*is the total number of principal components. This quantification is essential for determining the significance of each principal component in explaining the variance.

The results of PCA as seen in the Supplementary document Figs. S1, S2, and S3, provide critical insights into the most significant patterns within the data, enabling a more strong clustering process and provides a detailed comparison between modeled and empirical tourist flows.

### Simulating clustering behavior under perturbations

To evaluate the stability of the clustering results under small perturbations, we apply a Monte Carlo simulation framework to the transformed similarity matrix of the mobility flux. This approach allows us to evaluate the sensitivity of cluster assignments to minor variations, which may reflect realistic fluctuations.

Let $\textbf{S}$ represent the original matrix of tourist flows between countries, where each element $S_{ij}$ denotes the observed flow between country *i* and country *j*. To assess similarity, we first transformed $\textbf{S}$ using a distance metric, such as the Euclidean distance, to obtain a distance matrix $\textbf{D}$,

5 $$\begin{aligned} \textbf{D} = d(\textbf{S}), \end{aligned}$$

where $d(\cdot )$ represents the transformation operation applied to the mobility flux.

After obtaining the distance matrix $\textbf{D}$, we introduced a small perturbation to simulate potential variations in similarity due to factors such as seasonal fluctuations or measurement noise. The perturbed distance matrix, denoted by $\tilde{\textbf{D}}$, is given as,

6 $$\begin{aligned} \tilde{\textbf{D}} = \textbf{D} + \textbf{P}, \end{aligned}$$

where $\textbf{P}$ is a perturbation matrix with elements $P_{ij}$ drawn from a normal distribution with mean and variance matching those of $\textbf{D}$,

7 $$\begin{aligned} P_{ij} \sim \mathcal {N}(\mu _{D}, \sigma _{D}^2), \end{aligned}$$

where $\mu _{D}$ and $\sigma _{D}^2$ are the mean and variance of the original distance matrix $\textbf{D}$. This ensures that the perturbations reflect the inherent variability in the transformed data.

#### Monte Carlo simulation for stability evaluation

To assess the clustering stability, we generated $N = 100,000$ perturbed matrices $\tilde{\textbf{D}}_1, \tilde{\textbf{D}}_2, \dots , \tilde{\textbf{D}}_{N}$, each representing a plausible variation of the distance structure due to random fluctuations. For each perturbed matrix $\tilde{\textbf{D}}_n$, we perform agglomerative clustering to obtain a set of cluster labels $\{L_i^{(n)}\}$ for each country *i*.

The clustering stability is then evaluated by computing the silhouette score $s^{(n)}$ for each experiment *n*, given as,

8 $$\begin{aligned} s^{(n)} = \frac{1}{M} \sum _{i=1}^{M} \frac{b_i^{(n)} - a_i^{(n)}}{\max (a_i^{(n)}, b_i^{(n)})}, \end{aligned}$$

where *M* denotes the total number of countries, $a_i^{(n)}$ is the mean intra-cluster dissimilarity for country *i*, and $b_i^{(n)}$ is the lowest mean dissimilarity between *i* and any other cluster.

The silhouette scores are averaged over all simulations to provide an overall measure of clustering consistency,

9 $$\begin{aligned} \overline{s} = \frac{1}{N} \sum _{n=1}^{N} s^{(n)}. \end{aligned}$$

#### Aggregation of cluster labels and final visualization

To identify a stable clustering structure, the cluster labels $\{L_i^{(n)}\}$ are aggregated across all simulations by calculating the mode of the labels for each country *i*,

10 $$\begin{aligned} \hat{L}_i = {\text {mode}}(L_i^{(1)}, L_i^{(2)}, \dots , L_i^{(N)}), \end{aligned}$$

yielding a final cluster assignment $\hat{L}_i$ that reflects the most consistent grouping across perturbed matrices.

Additionally, we compute the average of the perturbed distance matrices, given as,

11 $$\begin{aligned} \overline{\tilde{\textbf{D}}} = \frac{1}{N} \sum _{n=1}^{N} \tilde{\textbf{D}}_n, \end{aligned}$$

and apply principal component analysis (PCA) and dendrogram visualization to this averaged matrix to capture the primary clustering patterns under realistic conditions of data variability.

## Results

### Stabilization and cohesive clustering

Table 3 presents the clustering performance for both empirical data and the model across the years 2019, 2020, and 2021. The table reports the highest silhouette score achieved by the corresponding clustering method, alongside the average silhouette score ($\text {MC Score}$) and standard deviation ($\sigma$) from 100,000 Monte Carlo simulations with perturbations.

**Fig. 2:**
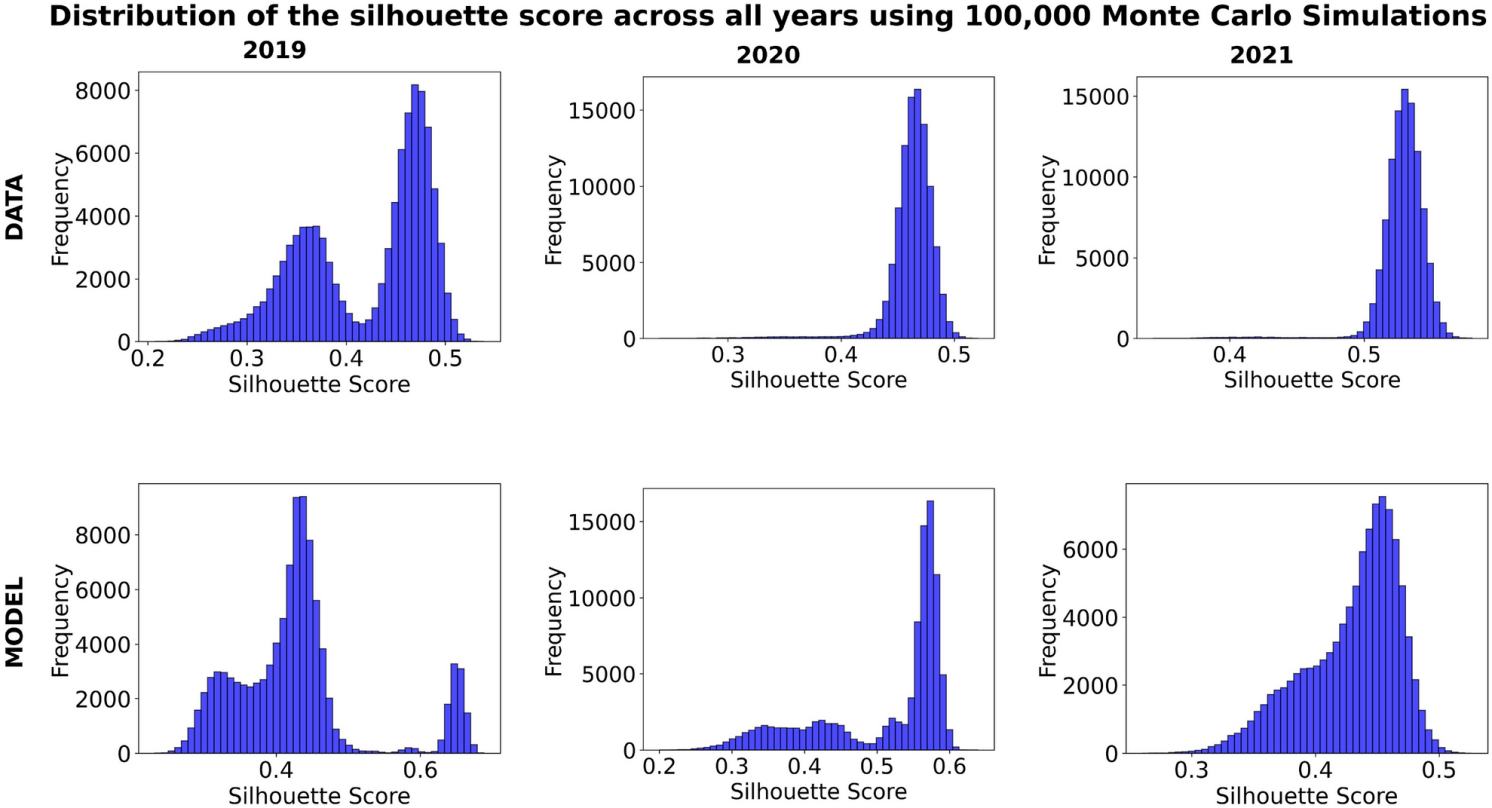
Distribution of silhouette scores across 100,000 Monte Carlo simulations with perturbations where the histograms illustrate the variability in silhouette scores for both the data and model across different years. The distributions reflect the stability and clustering performance under perturbations.

For 2019, the highest silhouette score achieved by the Agglomerative Clustering (Euclidean distance) was 0.743 for the empirical data and 0.769 for the model. However, the average silhouette scores obtained from the Monte Carlo simulations were significantly lower at 0.419 (data) and 0.428 (model), with corresponding standard deviations of 0.065 and 0.093. The larger standard deviations seems to reflect greater variability in clustering quality under perturbations. This is consistent with the bimodal distributions observed in the distribution of the silhouette scores as shown in Fig. 2, indicating the clustering results alternated between distinct quality levels.

The higher variability in the modeled data compared to the empirical data suggests that the model is more sensitive to perturbations for this year, potentially due to structural heterogeneity or noise amplification in the clustering process. It is vital to state that the bimodal behavior may reflect the presence of two distinct clustering regimes in both the empirical and modeled data where the bimodality suggests that the data contains two subgroups of countries; one subgroup where clustering is well-defined with higher silhouette scores, and another subgroup where clustering is weaker or more ambiguous where the silhouette scores are lower.

In 2020, the highest silhouette scores improved to 0.786 for the empirical data and 0.799 for the model, using Agglomerative Clustering with Manhattan distance (k=2). The average silhouette scores from the Monte Carlo simulations were 0.463 (data) and 0.511 (model), showing higher clustering stability compared to 2019. For the data the standard deviation decreased to 0.021 which may reflect a more consistent clustering performance under perturbations. However for the model the standard deviation improved slightly to 0.090 which reflects a greater variability in clustering which can be seen in the bimodal distribution of the silhouette scores.

For 2021, the empirical data and the model both achieved their highest silhouette scores using Agglomerative Clustering (Euclidean distance), with values of 0.841 and 0.784, respectively. The average silhouette scores from the Monte Carlo simulations were 0.529 (data) and 0.430 (model), with standard deviations of 0.017 and 0.039, respectively. The low standard deviations may indicate high clustering stability and minimal sensitivity to perturbations like the 2020 empirical data. This aligns with the unimodal distributions observed in the histograms for 2021, where the clustering results were consistent and did not vary across simulations. The empirical data achieved the highest average silhouette score across all years, further emphasizing its stability and well-defined clustering structure.

The results in Table 3 align well with the PCA visualizations and silhouette score distributions. Despite the variability observed in silhouette scores, the global clustering structure seems to remains sturdy, as evidenced by the Monte Carlo PCA plots and consistent Monte Carlo dendrograms across all years which remain unchanged when compared to the outcomes where perturbations have not been implemented as shown in Figs. S1, S2, and S3.

### Effects of perturbations on clustering stability and distribution patterns

Note that, for 2019, the clustering variability reflected in the bimodal silhouette score distribution is less apparent in the PCA, highlighting that global patterns are preserved even when local clustering quality varies. In 2020 and 2021, both PCA and silhouette score results confirm the increasing stability of clustering structures in both the model and empirical data. While variability in 2019 highlights potential data heterogeneity or noise sensitivity, the progressively stable results in 2020 and 2021 emphasize the reliability of the methodology, especially when applied to more homogeneous data structures. The Monte Carlo simulations demonstrate that despite perturbations, the global clustering structure is preserved, offering confidence in the strength of the methodology.

**Fig. 3:**
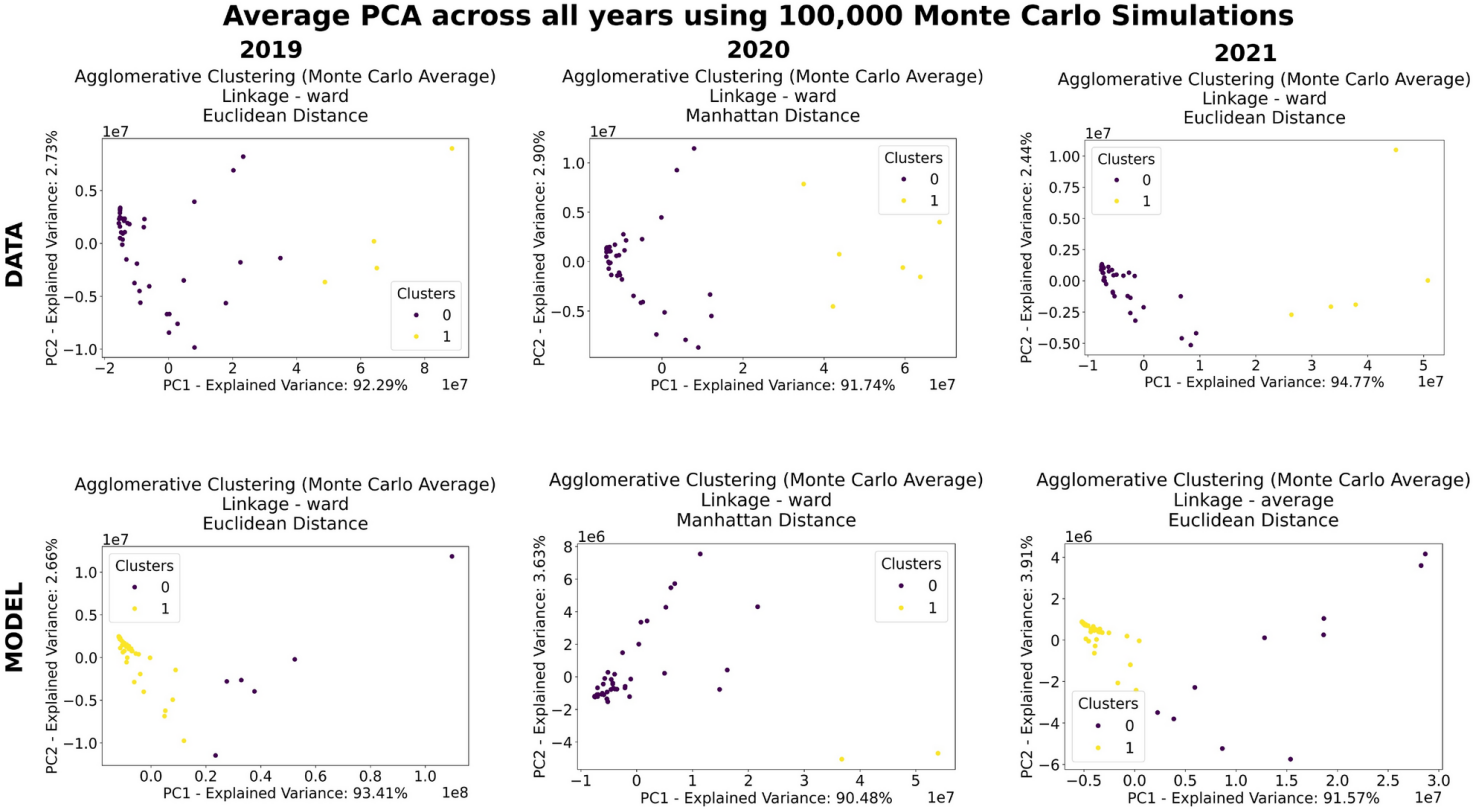
Using 100,000 Monte Carlo simulations, the figure shows the average PCA for the best clustering across all years for the data and the model.

The PCA results from the Monte Carlo simulations are presented in Fig. 3 which provide insights into the clustering dynamics of countries based on tourist behavior. This captures the primary dimensions of variability in the data for the years 2019, 2020, and 2021. These visualizations complement the silhouette score distributions and helps shed light on the patterns and the stability of the clustering under perturbations across different years.

For 2019, the PCA plots show a high proportion of variance explained by the first principal component (PC1), with 92.29% for empirical data and 93.41% for the model. This high explained variance indicates that the most significant dimension of variability in tourist behavior is concentrated in a few dominant factors. These factors likely include strong economic or cultural ties between countries, which drive consistent and predictable tourist flows.

The empirical data shows a concave upward trend, indicating that while the clustering is stable, there is some distribution of tourist behavior across countries, reflecting heterogeneous patterns among less dominant regions. The model, on the other hand, exhibits a stronger concave upward trend, likely reflecting a generalization of tourist flows, where the clustering method captures broader patterns in global mobility. The difference in curvature implies that the empirical data contains more localized or region-specific tourist behavior, while the model aims to capture more generalized global trends.

In 2020, the PCA plots reflect the disruption caused by the COVID-19 pandemic on tourist mobility. The variance explained by PC1 is reduced to 91.74% for empirical data and 90.48% for the model, indicating a breakdown in the usual dominant patterns of tourism behavior. This reduction suggests that traditional economic and cultural corridors were weakened and restructured during the pandemic, with new factors emerging to influence tourist flows.

The PCA results explain how the pandemic forced changes in mobility patterns and sheds light onto the fragility of socio-economic networks that rely heavily on tourism. Therefore the PCA plots for 2020 show a wider spread of data points along PC1 and PC2, particularly for the empirical data. This increased spread suggests greater variability in tourist behavior, consistent with the changes in silhouette scores observed for this year. The dispersal of points suggests that tourist behavior became less predictable, with countries diverging from their typical clustering patterns. The pandemic’s impact likely forced tourists to shift towards domestic or regional travel, disrupting established travel routes and leading to less cohesive clusters.

### Distance metrics and clustering performance

The unique characteristics of the 2020 tourist mobility data, heavily influenced by the COVID-19 pandemic, introduced significant outliers and sparsity due to the abrupt and uneven reductions in tourist flows. Consequently, the preferred distance metric for the empirical data and model in 2020 differed from other years. HAC was applied using the Manhattan distance, which outperformed the Euclidean distance by achieving higher silhouette scores. This advantage can be attributed to the Manhattan distance’s reliance on summing absolute differences, which makes it less sensitive to outliers and ensures that all dimensions are treated equally.

The Manhattan distance proved to be particularly well-suited for capturing the overall patterns of change across all country pairs without allowing extreme values to disproportionately influence the clustering. Unlike the Euclidean distance, which squares differences and amplifies the impact of outliers (e.g., missing UK data), the Manhattan distance minimizes the risk of misleading cluster formations. During periods of significant deviation from normal patterns, such as the pandemic, it is crucial to treat all dimensions equally. This aligns with the need to uniformly consider all tourist flow changes, further highlighting the importance of using the Manhattan distance in handling the the 2020 empirical data and model.

By 2021, both the empirical data and the model show concave upward trends as shown in Fig. 3, with variances explained by PC1 at 94.77% for empirical data and 91.57% for the model. These high explained variances indicate that tourist behavior largely stabilized, with clustering becoming more stable and cohesive which is indicative by the narrower spread of points along PC1 and PC2.

The concave upward trend in both datasets suggests that tourist flows became more centralized around key destinations, reflecting a recovery in global mobility patterns. The similar curvature between empirical and modeled data in 2021 indicates that the model is now closely aligned with observed tourist behavior, suggesting that the recovery was predictable and well-captured by the clustering methodology. The stabilization of PCA shapes and the high variance explained by PC1 suggest that countries returned to their pre-pandemic clustering structure, though some residual effects of the pandemic may still influence smaller variances in PC2.

Similar to 2019, the empirical data for 2021 shows a concave upward trend, suggesting the dominance of a few key hubs or corridors driving tourist flows. The modeled data, while slightly less centralized, continues to reflect strong clustering patterns, capturing global mobility trends while maintaining the overall clustering structure.

In terms of linkage for the 2021 model, average linkage emerged as the preferred method over ward linkage which was preferred for the 2019 and 2020 empirical data and 2019 model. Average linkage’s ability to produce more evenly distributed data points resulted in well-separated and compact clusters, leading to a higher silhouette score. In contrast, ward linkage, which emphasizes compactness over separation, tended to form overly tight clusters that were less distinct, resulting in lower clustering performance for the 2021 modeled data. Using average linkage allowed the distribution of the silhouette scores as shown in Fig. 2 maintains a similar shape to its empirical data counter part in terms of being unimodal but with a right tail. It must be noted that this right tail behavior in the distribution is exhibited as well in the 2020 model as well which could have had some adverse effects.

**Fig. 4:**
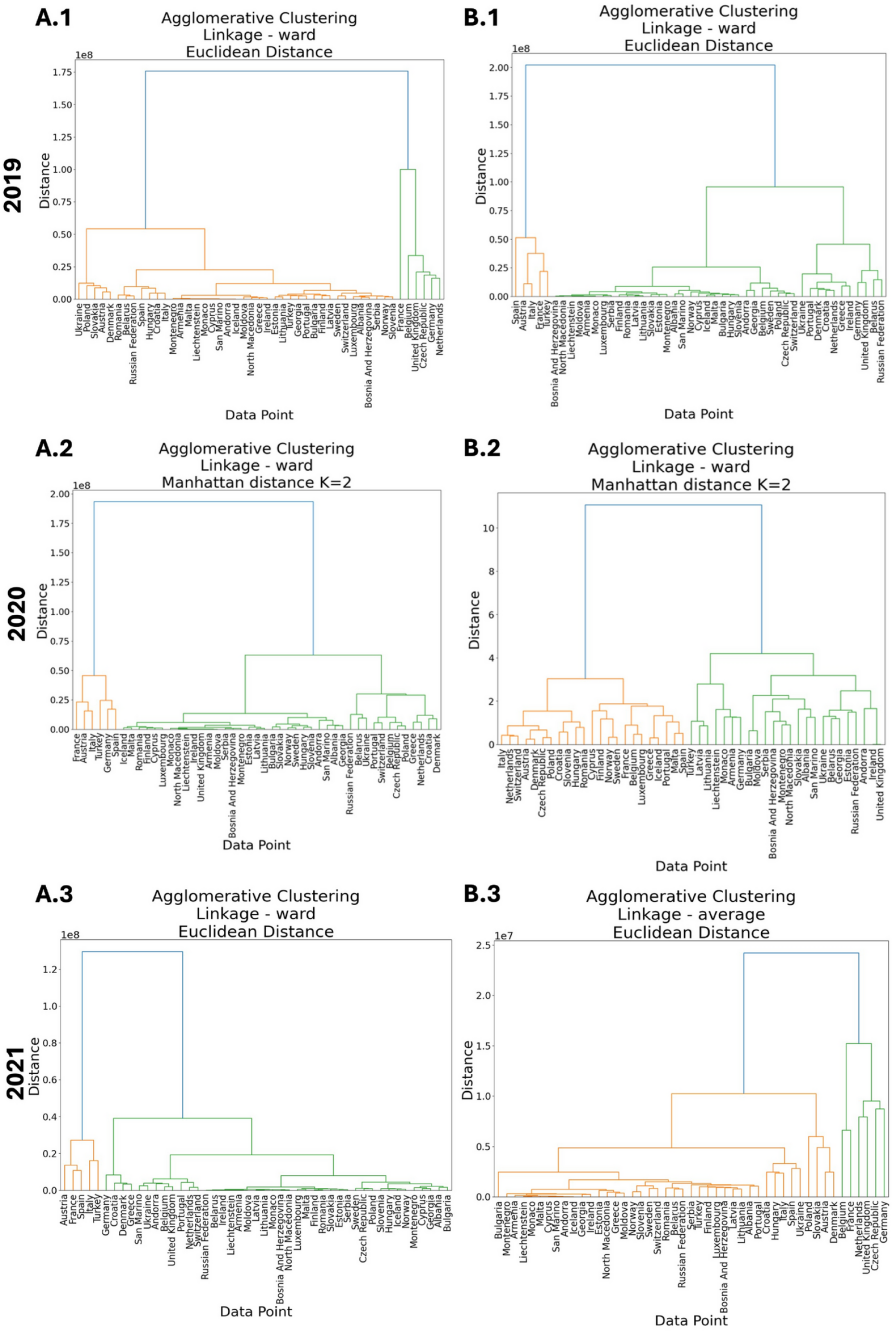
Dendrogram visualizations based on the average mobility flux after 100,000 Monte Carlo simulations. (**A.1–A.3**) The dendrogram for the best clustering method that has the highest silhouette score for the data for all years. (**B.1–B.3**) The dendrogram for the best clustering method that has the highest silhouette score for the modeled data for all years.

Given that HAC produces a tree-like structure, the dendrogram presented in Fig. 4 illustrates the hierarchical clustering process, revealing the clusters at varying levels of granularity. This visual representation provides further insights into the structural differences in clustering behavior across the years. Importantly, these dendrograms were generated after running multiple Monte Carlo (MC) simulations, which incorporated perturbations into the distance matrices to evaluate the strength and stability of the clustering process. They show that the dendrograms produced after running Monte Carlo simulations do not differ from the dendrograms produced without any perturbations added.

The Monte Carlo simulations involved creating 100,000 variations of the distance matrices by introducing small amounts of noise, designed to have the same mean and variance as the original data. This process was used to reflect potential variability in tourist behavior, such as inconsistencies from missing values, measurement errors, or random fluctuations in tourist flows. Clustering was performed on each perturbed matrix, and the results were aggregated to evaluate how consistently clusters emerged across different scenarios.

By averaging the perturbed matrices, the dendrogram reveals the most stable and strong clustering structure, highlighting patterns that persist even under varying noise conditions. This approach helps identify the core clusters that remain consistent despite the natural variability in the data, adding confidence to the reliability of the results. The branch lengths and levels in the dendrogram further illustrate the granularity of clustering across different years, providing insights into how tourist mobility patterns have shifted and how stable these patterns are when subjected to noise. The Monte Carlo framework ensures that the clustering outcomes are not just artifacts of data imperfections but reflect meaningful and resilient groupings of countries based on tourist behavior.

The significant reduction in the average silhouette score after running MC simulations, compared to the silhouette score produced without perturbations, highlights the impact of variability and noise on clustering quality. However, the fact that the clusters formed remain the same suggests that the global clustering structure is very strong, even when the data is subjected to noise and perturbations.

This discrepancy between silhouette scores can be attributed to the sensitivity of the silhouette score to local variations in the data. Perturbations introduced during MC simulations likely distort some inter-cluster and intra-cluster distances, leading to lower silhouette scores. Despite this, the clustering algorithm successfully preserves the core structure of the clusters, as evidenced by the consistent groupings seen in the PCA plots and dendrograms. This implies that while the noise may affect local clustering metrics, it does not disrupt the overall partitioning of countries into meaningful clusters.

### Data handling

The results also emphasize the impact of missing data, particularly the absence of tourist flow data from the United Kingdom. The exclusion of the UK reduces the data size and increases sparsity, which amplifies the effect of outliers when using the Euclidean distance. This limitation is particularly pronounced in 2020, where the sparsity and irregularities in the dataset affected clustering outcomes.

To address this issue, the Manhattan distance was used instead, as it is less sensitive to outliers and irregularities. By summing absolute differences, the Manhattan distance ensured a stronger clustering process despite the challenges posed by the missing UK data. This adjustment allowed for the preservation of meaningful cluster structures, maintaining the integrity of the analysis and providing a clearer picture of tourist mobility during this period. The data handling protocol is discussed in detail in the Supplementary file.

We note that the exclusion of the UK likely contributed to the observed differences in clustering performance and metric effectiveness. Recognizing this limitation is crucial for interpreting our results accurately. Future studies should aim to include comprehensive data from all major countries to enhance the strength of the model.

## Discussion

This study highlights the fragility and resilience of Europe’s socio-economic corridors, particularly in the face of global disruptions like the COVID-19 pandemic. By integrating perturbations into the clustering and PCA analyses, our work provides a unique perspective on the structural dynamics of tourism-driven economic systems. One significant finding is the sensitivity of tourist mobility patterns to socio-economic disparities, as evidenced by the Monte Carlo clustering and dendrogram analyses (Fig. 4). These results highlight the tendency of tourists to prefer destinations with similar GDPs, cultures, and languages, reinforcing regional cohesion while exposing vulnerabilities to external shocks.

By introducing controlled noise into the distance matrices and running Monte Carlo simulations, we evaluated the strength of clustering under variability. This method allowed us to identify stable patterns in tourist mobility, even in the presence of inconsistencies or data gaps. The ability to capture strong clustering structures despite perturbations provides a more reliable foundation for understanding the dynamics of socio-economic networks and offers policymakers confidence in the results.

The absence of UK tourist flow data, a notable limitation in this study, likely influenced clustering outcomes and the effectiveness of certain metrics. The UK plays a pivotal role in Europe’s socio-economic landscape, particularly within the Blue Banana economic corridor, a densely populated and economically prosperous region that anchors Europe’s economic structure^7^. The exclusion of this data amplifies sparsity and increases sensitivity to outliers, necessitating adjustments such as the use of Manhattan distance in 2020, which proved more strong under these conditions. Therefore addressing data gaps and inconsistencies we introduced perturbations to assess the stability and adaptability of the clustering.

The 2006 policy landscape of the European Union significantly contributed to the development and strengthening of economic corridors across the continent. Through the revision of TEN-T, the Lisbon Strategy, the European Cohesion Policy, and the ETC Framework, the EU set the stage for a more integrated and interconnected economic area^8,19^. These policies collectively ensured that transport and economic infrastructure were enhanced to support both the established corridors like the Blue Banana and emerging corridors in Southern and Eastern Europe, thereby promoting unity and cohesion across the EU.

It is important to keep in mind the UK’s influence in Europe due to its major contributions to socio-economic dynamics. Van der Meer has emphasized the importance of integrated economic corridors that enhances European development. This study shows the fragility of the system and shows that there needs to be improvements introduced to strengthen the economic corridors so that all parts of the Europe develop equally. We shows that some areas of Europe are susceptible to isolation and exclusion during a crisis. Consequently, COVID-19 exposed the vulnerabilities of global and regional economic systems which requires policyholders to take action.Noting that the UK is a central player in the Blue Banana economic corridor^7^, we highlight the need to add perturbations to overcome the limitation of missing data.

To diversify the Blue Banana and ensure the Red Octopus economic corridor does not fall apart again, small and medium sized enterprises (SMEs) should be encouraged as they are key drivers in innovation and economic growth which aids GDP growth^9,54^. By taking the UK as an example of SME investment during the COVID-19 pandemic, the UK government recognized the critical role that SMEs play in the economy^55^, as they represent a large portion of employment and contribute significantly to GDP. Therefore they provided many schemes such as the Job Retention Scheme (furlough), Small Business Grants Fund (SBGF), Coronavirus Business Interruption Loan Scheme (CBILS), etc to aid economic recovery after the pandemic^56^. It provided liquidity, prevented business closures, and helped retain jobs, demonstrating that much of the government’s assistance indeed targeted SMEs which allowed the UK to bounce back and return as a key economic player in norther Europe as outlined in Fig. 1.

From an economic perspective, our findings explain the fragility of emerging corridors like the Red Octopus, which, during times of distress, revert to the stability of the Blue Banana corridor. This reversion highlights the need for targeted policy measures to strengthen less resilient regions and ensure balanced development across Europe. The results also emphasize the critical role of small and medium-sized enterprises (SMEs) in fostering resilience and economic recovery. For example, during the COVID-19 pandemic, the UK’s targeted support for SMEs through initiatives such as the Job Retention Scheme and Small Business Grants demonstrated how effective policies can mitigate disruptions and enable rapid recovery^55,56^.

Moreover, the MC PCA results, seen in Fig. 3, and the distribution of silhouette scores, seen in Fig. 2, further highlight the structural changes in tourist mobility over time, offering a tool for policymakers to anticipate and respond to shifts in tourism flows. For instance, the concentrated clustering patterns observed in 2021 suggest a stabilization of tourist behavior, with countries either recovering or maintaining consistent patterns. However, the persistence of distinct clusters and preferences for intra-regional travel, as seen in the MC dendrograms, Fig. 4, indicates that tourists favor destinations with similar socio-economic and cultural characteristics. This preference aligns with the clustering results, which emphasize the role of GDP, language, and cultural proximity in shaping mobility patterns.

It highlights the structural changes and explains the importance of strengthening the unity between countries in the socio-economic corridors as outlined by the Red Octopus^10,18,57^. To remain a key player, firstly high value should be placed in SMEs^56^ as it also boosts domestic consumption and also the transport infrastructure between countries should be revisited.

These findings are particularly relevant for reinforcing the socio-economic unity of Europe. Policymakers should prioritize strengthening transport infrastructure, such as railways and airports, to enhance connectivity between regions. Additionally, focusing on smaller clusters and targeting tourists from similar cultural and economic backgrounds can optimize marketing strategies and improve resource allocation. These actionable insights are especially critical for less developed regions within the Red Octopus corridor, which require targeted investment to match the resilience of the Blue Banana corridor. Consequently, on a smaller scale the actionable points that requires less funds would be to target their finer clusters as shown in the dendrograms. Whereas actionable points would be to promote SME growth and strengthen transport infrastructure to reinforce socio-economic corridors.

The silhouette score distributions reveal key trends in tourist behavior and clustering performance across the three years. In 2019, the bimodal distribution highlights the heterogeneity of tourist behavior, possibly driven by diverse regional patterns, varying levels of tourism development, or external factors like economic conditions. This variability suggests two distinct clustering regimes, with one group exhibiting well-defined clusters and another showing more ambiguous patterns. The larger spread of scores indicates sensitivity to perturbations and underlying disparities in mobility dynamics.

In contrast, the distributions for 2020 and 2021 are unimodal, reflecting greater uniformity and stability in clustering performance. For 2020, the narrower histogram suggests high consistency in tourist behavior globally, likely a result of universal travel restrictions and health concerns during the pandemic. By 2021, the distributions become even more concentrated, with peaks indicating well-defined clusters and minimal noise. This progression highlights a convergence in tourist behavior patterns and a recovery toward pre-pandemic stability. The alignment of distributions between empirical and modeled data in 2021 suggests that the model effectively captures the recovery trajectory, though minor differences in peak scores reflect subtle deviations between observed and modeled patterns.

Therefore, this study establishes work for geo-social-spatial modeling by conceptualizing destinations as polygons, allowing for the application of spatial analysis techniques to evaluate the model’s effectiveness in geo-social-spatial contexts. We treat destinations as single monomer sources of radiation. The current framework may overlook regional variations in tourist mobility and introduce biases, particularly when capital cities are not centrally located within their respective countries. Additionally, the assumption that countries are perfectly spherical contributes to the simplification of the model, necessitating the use of capital cities as proxies for entire countries.

Consequently, to address these limitations, a potential improvement involves extending this framework to model destinations as polymers composed of multiple weighted monomers, each with varying levels of radiation. This approach would allow for a better distribution of radiation along borders, enhancing spatial resolution by incorporating multiple weighted central points. However, implementing this improvement would require more detailed data on the spatial arrangement and characteristics of the individual monomers.

## Supplementary Information

Supplementary Information.

## Data Availability

The data that support the findings of this study are available from the World Tourism Organization’s Yearbook of Tourism Statistics but restrictions apply to the availability of the data as they need to be purchased. Source code available at https://www.github.com/fmuk23/Covid19-Migration-Patterns/.
