# Supplementary material for: Machine learning analysis of the effects of COVID-19 on migration patterns
Source: Sci Rep. 2024 Nov 30;14:29815. doi: 10.1038/s41598-024-80841-0 (PMC11608230; doi:10.1038/s41598-024-80841-0)
Supplement: Supplementary file 1 — Supplementary Information. [file 41598_2024_80841_MOESM1_ESM.pdf]

Machine learning analysis of the effects of COVID-19 on migration patterns: Supplementary document

FARZONA MUKHAMEDOVA[1] AND IVAN TYUKIN[1]

[1] King's College London, London, WC2 R2LS, United Kingdom

This supplementary file provides information on the data handling protocol and the results of the principal component analysis.

1. DATA HANDLING PROTOCOL

The following is a supplementary guide with more detailed instructions on how to use the code with the available data.

We report that the missing data within the study is handled as no movement where there is no migration from the source to the destination. In the data acquisition process arrivals are considered non-resident visitors at national borders unless this information is not available. In cases where such data is not provided, the classification follows this order: arrivals of non-resident tourists at national borders, arrivals of non-resident tourists in all types of accommodation establishments, arrivals of non-resident tourists in hotels and similar establishments, overnight stays of non-resident tourists in all types of accommodation establishments, overnight stays of non-resident tourists in hotels and similar establishments.

The provided code can be enhanced with additional migration data as it becomes available in the future. The current setup is designed for one year but is repeated for subsequent years, as the framework is established for both the model and data. All distance metrics and clustering algorithms are evaluated independently to make it user friendly. All images and metrics are produced within this code as well as metrics such as elbow points used in k-means clustering to identify the optimal k to use. The silhouette scores as well as the clustering results are saved onto Excel files automatically.

The inputs required are clearly outlined in the accompanying code in the given repository. The code requires the latitude and longitude of the capital city or the geographic center of the country the user wishes to represent. It also needs population data for each country, along with information on the total number of people migrating from the source to the destination. These inputs are explicitly stated within the code. The synthetic model used to replicate migration patters is also explicitly outlined in the code that is used for executing the analyses. This model in future works can be applied to different migration patterns such as the migration seen at different border checkpoints, counties within a country or even modified to include polygon shapes.

To promote a user-friendly method, transparency and reproducibility, we have included this comprehensive supplementary guide that details the analytical methods and provides instructions for using our code with alternative datasets. Researchers can adapt the code to their own data by following the data formatting guidelines and adjusting parameters as

described. We encourage the research community to engage with our work through the GitHub repository or by contacting us directly at farzona.mukhamedova@kcl.ac.uk.

2. RESULTS OF THE PCA

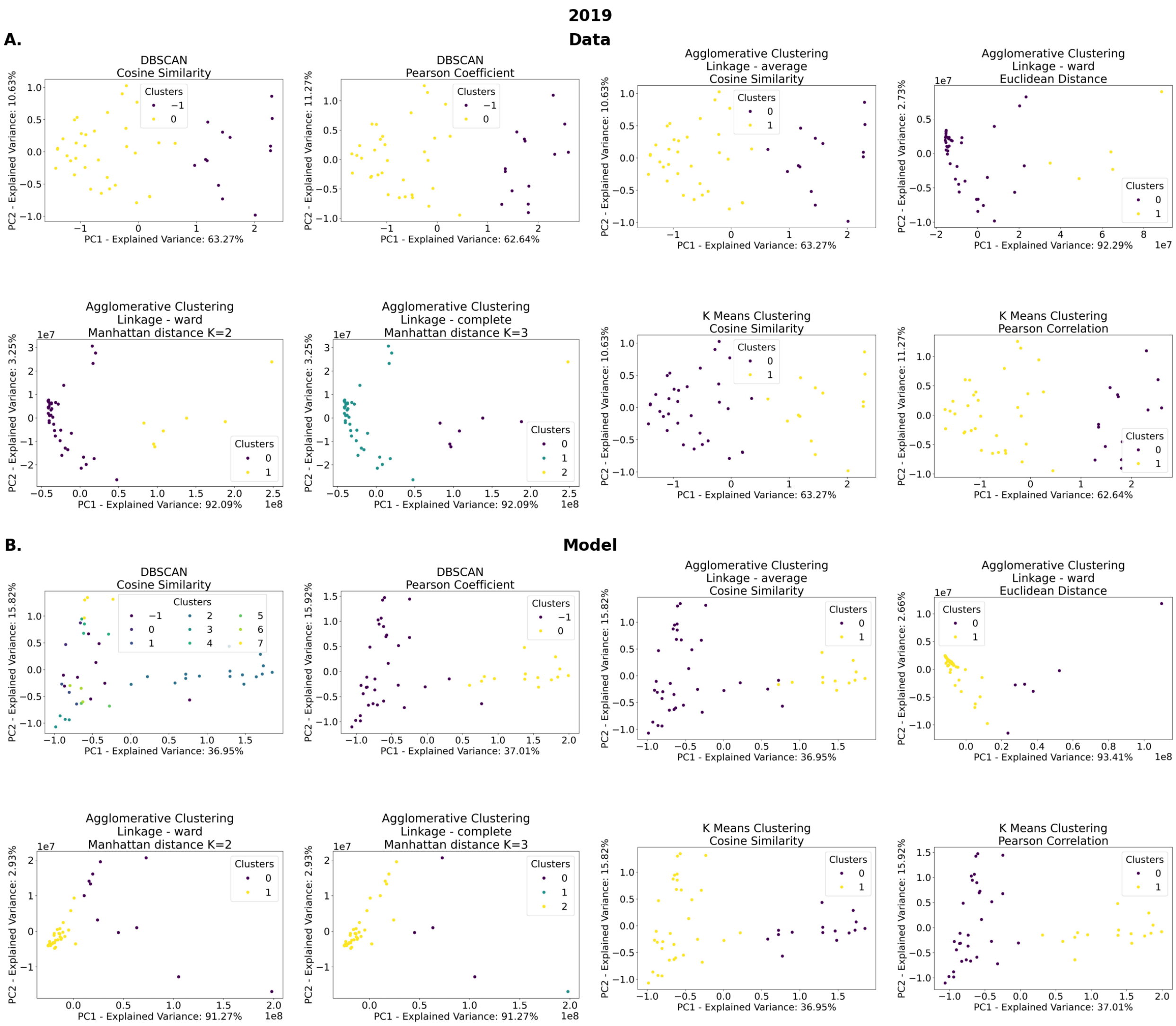

Fig. S1. Principal component analysis of the different unsupervised machine learning models coupled with similarity measures for the model and data from 2019.

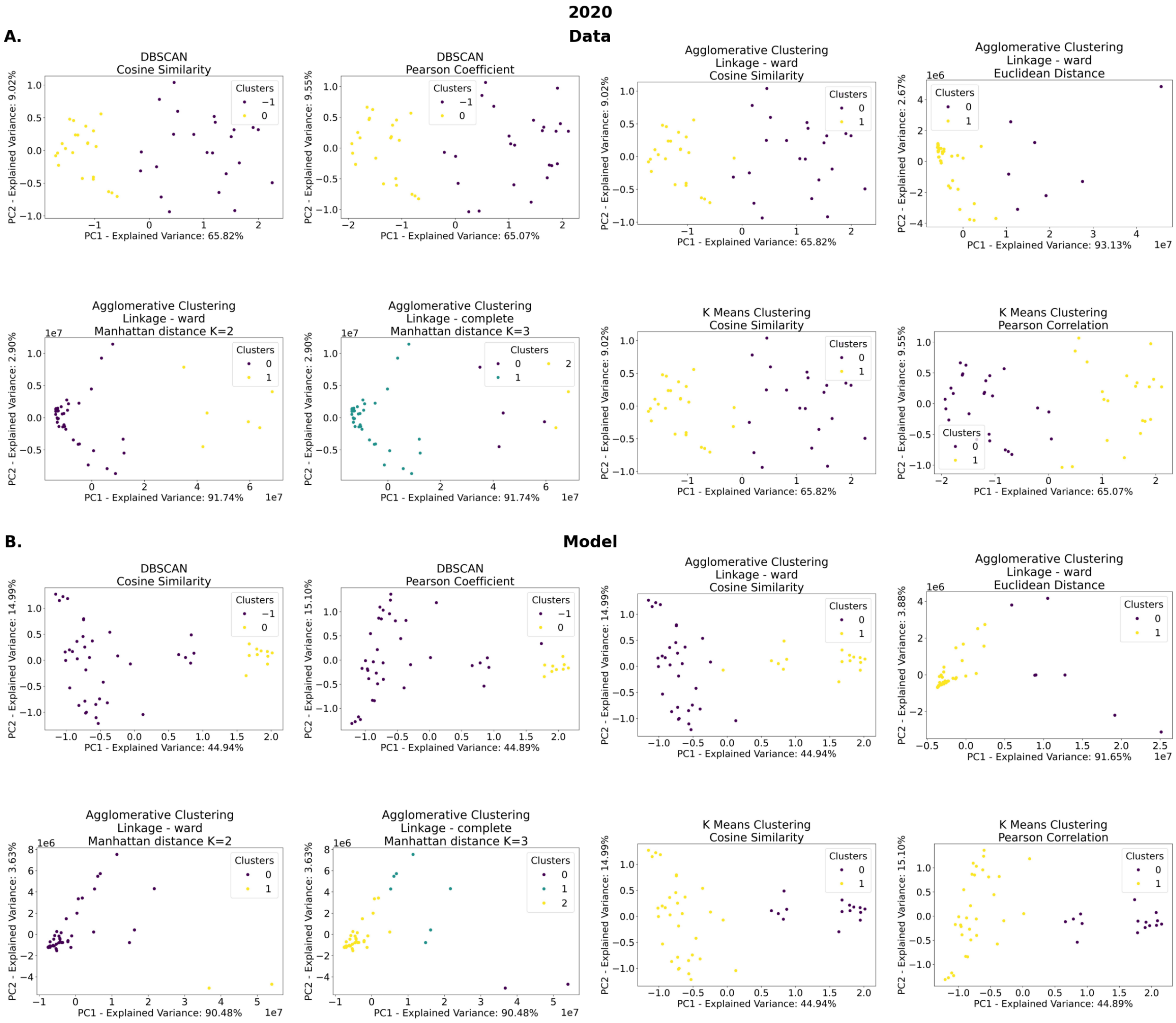

Fig. S2. Principal component analysis of the different unsupervised machine learning models coupled with similarity measures for the model and data from 2020.

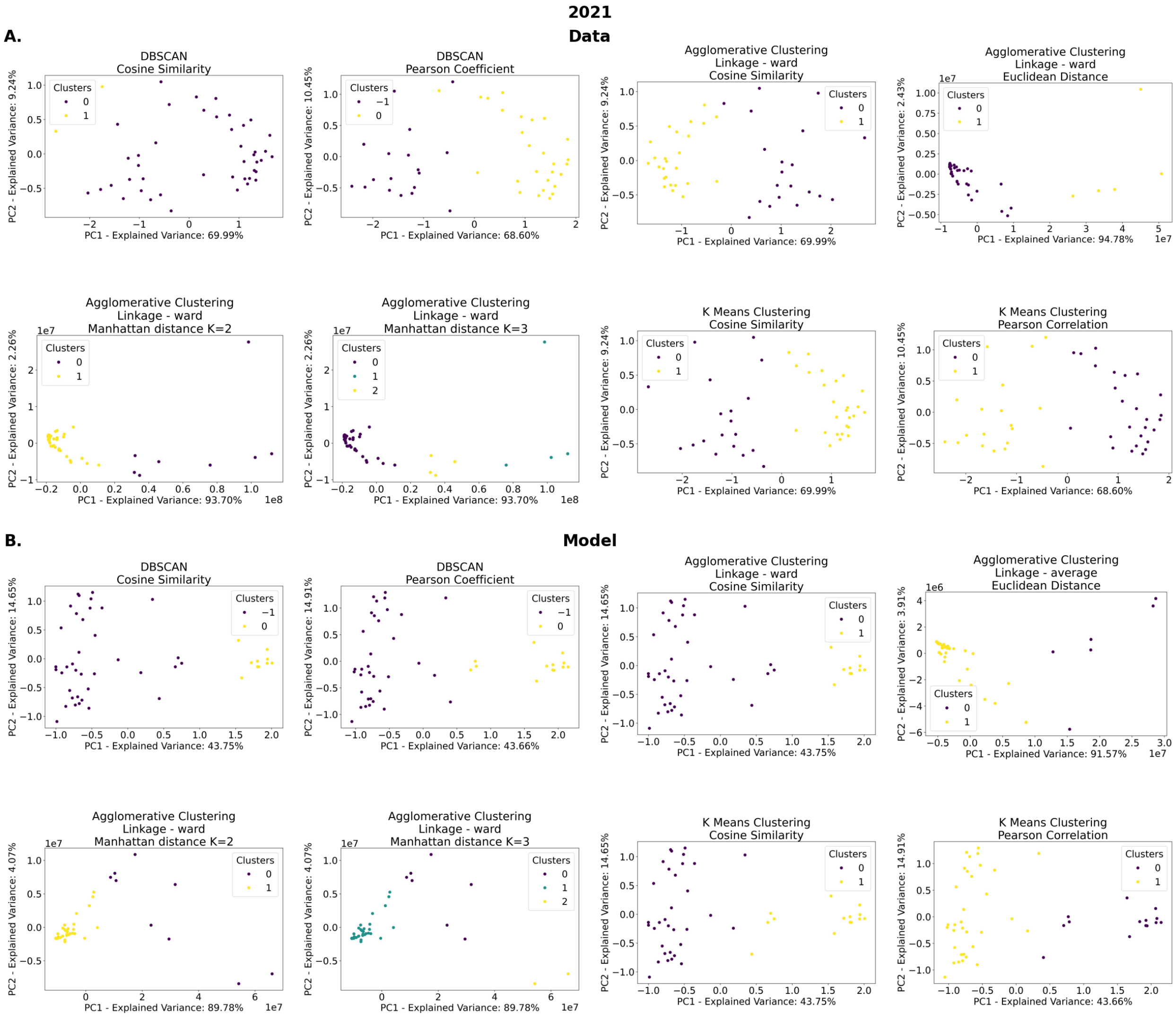

Fig. S3. Principal component analysis of the different unsupervised machine learning models coupled with similarity measures for the model and data from 2021.
